# Conducting practice-based projects among chiropractors: a manual

**DOI:** 10.1186/2045-709X-21-8

**Published:** 2013-02-01

**Authors:** Iben Axén, Charlotte Leboeuf-Yde

**Affiliations:** 1 Karolinska Institutet, Unit of Intervention & Implementation Research, Institute of Environmental Medicine, Nobels väg 13, S-171 77, Stockholm, Sweden; 2 Research Department, Spinecenter of Southern Denmark, Institute of Regional Health Research, University of Southern Denmark, Hospital Lillebælt, Østre Hougvej 55, DK-5500, Middelfart, Denmark

**Keywords:** Clinical study, Compliance, Multicentre

## Abstract

**Introduction:**

Practice-based research is a challenge as clinicians are busy with their patients and any participation in research activities will be secondary to the needs of the patients and the clinic. As a result, it is difficult to obtain high compliance among clinicians. A method to enhance compliance in multicentre practice-based research has been developed and refined for use in the chiropractic setting and possibly also by other researchers in different settings.

**Method:**

This manual provides a stringent step-by-step approach for conducting clinic-based research. It describes the competencies and requirements of an effective working group, how to recruit participating clinicians and how to empower, encourage and support these clinicians to obtain good compliance.

**Discussion:**

The main advantage of the method is the high compliance of participating clinicians compared to many other clinical studies. Difficulties with the method are described and suggestions for solutions are presented.

**Conclusions:**

This manual is a description of a method that may be of use for clinical researchers in the chiropractic setting.

## Background

Clinical research has the advantage of mimicking clinical reality. The patient population, the procedures, the decisions and the outcomes will represent what happens normally. Further, the study results are easier to implement if the studied procedures are already tested in a clinical setting.

Over the past 15 years, a number of practice-based research projects have been performed among chiropractors in the Nordic countries in which data on patients have been collected using questionnaires [1-12]. Some of these studies are summarized in Table 1. In addition, an international study has been carried out [13]. The above studies have all been multi-centre clinical studies and they have yielded high compliance rates compared to similar research [14-16].

**Table 1:** Compliance rates in some studiescarried out using the herein described procedures

**Study title**	**Country**	**Year**	**Method**	**Number of included patients**	**Compliance, chiropractors**	**Compliance, patients**
**Chiropractic in Sweden: a short description of patients and treatment.**	Sweden	1997	Cross-sectional	628	78%	73%
**The types and frequencies of improved nonmusculoskeletal symptoms reported after chiropractic spinal manipulative therapy.**	Sweden	1999	Cross- sectional	1504	81%	86%
**The nordic back pain subpopulation program: demographic and clinical predictors for outcome in patients receiving chiropractic treatment for persistent low back pain.**	Norway	2004	Prospective outcome	875	56%	76%
**Can patient reactions to the first chiropractic treatment predict early favorable treatment outcome in persistent low back pain?**	Sweden	2002	Prospective outcome	615	74%	58%
**The Nordic back pain subpopulation program: can patient reactions to the first chiropractic treatment predict early favorable treatment outcome in nonpersistent low back pain?**	Sweden	2005	Prospective Outcome & predictive validity	674	86%	56%
**The Nordic Back Pain Subpopulation Program: validation and improvement of a predictive model for treatment outcome in patients with low back pain receiving chiropractic treatment.**	Sweden	2005	Prospective Outcome & predictive validity	1057	91%	61%

The successful completion of these projects depended on the participation of four groups of people: 1) the professional researchers, 2) the project group members, 3) the data collecting chiropractors, and 4) the patients.

The professional researcher(s) is/are responsible for the methodological aspects of the study, data analysis and the final report. The project group together with the researcher(s) assists both in the conceptual stages and the data collection. It may also be active during analysis and report preparation. The data collecting chiropractors are responsible for the data collection and the patients provide the data needed for the study.

Over the years, we have developed procedures to optimize the involvement of all these participants. The purpose of this report is to describe our work in detail. In other words, it can be considered a manual in practice-based research for chiropractors. We explain the importance of having a dedicated project group which is responsible for personal contacts with the data collecting clinicians, how this group should be selected and instructed and how this group should organize the execution of the study. We also provide instructions for dealing with the data collecting practitioners and how these should collect data from their patients. Finally, we describe how the work after data collection could proceed.

We believe that this method can be used to recruit and encourage chiropractors to participate in practice based research in other settings as well, even though it might be necessary to adapt somewhat to different cultural situations. It is likely that this procedure can be applied also to other professional groups.

## Methods

### The people involved

#### Researchers

**Figure 1:**
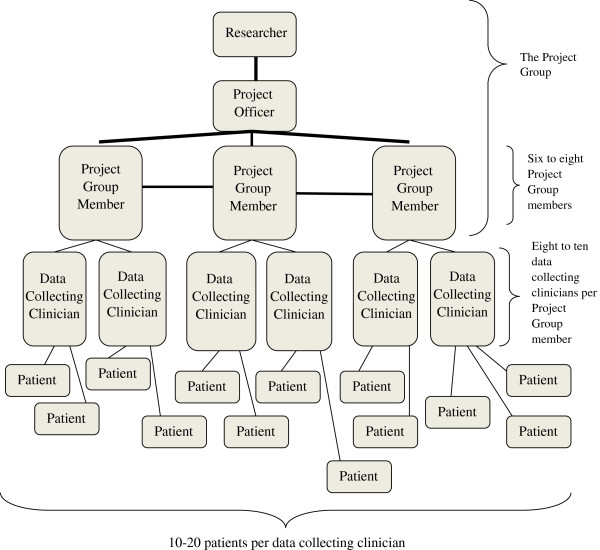
The organisational structure.

An overview of the organisational structure is found in Figure 1. One or several researchers may recruit collaborators for a study of a predetermined topic, or a group of clinicians with an interesting research question may enlist the assistance of one or several qualified (academic) researcher(s).

The researcher will act as the intellectual anchor of the project, the guarantor of the quality of method and the final report. If several researchers are involved in the project, it would be preferable to make use of people with different areas of expertise (knowledge of the literature, methodological experience or statistical expertise, for example).

The responsible researcher should be formally appointed in the early planning stages of a research project. This person should be familiar with the topic at hand. Usually, this is the person with the research idea, the one who “owns” the project, i.e. he/she is intellectually responsible for the research project.

The researcher has the final say on each stage of the research process and must keep a close eye on people, process and progress. This person must be easily available to deal with urgent issues arising during the study process.

#### Project group

Research should be clinically relevant. It is possible to enlist the participation of astute clinically active practitioners only if the research project they shall help with feels clinically relevant to them.

A project group composed of clinicians helps in formulating study goals and in the planning of the project and in data collection. A dedicated and active project group is of great help in the data collection stage, as its members are needed to obtain high compliance among the data collecting clinicians, as further described below. Active participation in each stage of the study promotes ownership for the project and good collaborators which will help improve the quality of data by minimizing errors in procedures and the handling of questionnaires. If the project group participates also in the data analysis, or at least in the data interpretation, and has a say in the manuscript phase, an added benefit is that their participation will enlighten them on the rigours of research and make them knowledgeable in this particular research area. Another advantage is that if several people donate their time, the onus of the work can be divided between several persons, which will reduce the need for funding.

The participants in the project group need not be qualified in terms of research competence, but being clinicians they will provide many invaluable viewpoints when preparing a study in a clinical setting. In fact, if the research leader is not a clinician, such clinical input is vital for the successful completion of the project. Clinicians can also foresee practical issues regarding time expenditure, delegation of tasks to receptionists, willingness of colleagues to donate the necessary time and suchlike.

Only truly dedicated clinicians should be recruited as project group members. Participation should be voluntary and based on a wish to do practice based research and to search for answers to clinically important questions. A letter from the research leader explaining the need for practice-based research and asking those interested to get in touch is a good way to start out but head-hunting may be a better alternative.

An introductory meeting will make it clear who would be willing to dedicate the necessary time and effort, and the first project group can be formed. “Interested” people who cannot make it to such introduction meetings should not be included in the group, as their first difficulty in participation often is indicative of their future level of involvement.

If several projects will follow, recruitment can probably take place by word of mouth. Project group participants may vary over time. Obviously, it is an advantage if the good co-workers stay on in the group, as people who are involved in several projects in this manner get proficient, making work in the project group increasingly smooth and effective.

Practice-based projects can, of course, be carried out without the help of an active project group. This does, however, require that the researcher (or a research assistant), who is responsible for the recruitment and continued follow-up of the data collecting practitioners, will have to work more or less full time over extended periods. This is probably possible only during a Ph.D. project or for a full-time employed researcher.

It is also possible to set up specific research clinics, whose clinicians will receive training in the data collection process and who enter into a contractual position with a researcher or a research institution, with or without financial compensation for their data collection activities. Obviously, if a formal “employment” exists, the situation is different and this manual is not totally relevant for such a set up.

#### Project officer

The researcher may not necessarily live or work locally close to the participants of the study. If not, it is necessary to appoint a project officer from within the project group. This is the person who is responsible for the logistics of the study and the only person within the project group who communicates directly with the researcher and he/she is also the person who ensures communication within the group.

The project officer must be particularly dedicated to the project, completely trustworthy, and very methodical in his/her approach to the task at hand. He/she must be respected and liked by colleagues; kind, courteous and positive, yet firm.

If funding is available, this person should receive remuneration for some or all of his/her work, as it is quite time consuming and will sometimes have to take place during clinic hours, which will result in a loss of income.

#### Data collecting participants

In practice-based studies, it is important to collect data from clinicians who are typical of their professional group. It is therefore important to make such “ordinary” clinicians interested in the project. Most clinicians are interested in the future of their profession, and many will agree to participate. In our studies, these clinicians will then be asked to collect standardized information from a number of patients who fulfill some specific inclusion criteria. The number of clinicians needed for the study will depend on the number of patients needed. This in turn depends on the study design. Our experience is that most clinicians will be willing and able to collect data on 10 patients, if the patient category is one frequently seen. If also no follow-up data are required (i.e. data are only collected at one point in time), more patients per clinician can be included (e.g. 20), as the logistics for the individual clinic is easier in such studies. However, the longer time the data collection will take for each patient and the more complicated the inclusion and/or the follow-up procedure, the more difficult it will be to obtain collaboration.

A high participation rate and valid data are key components of a successful data collection. To achieve this, it will be necessary to explain the purpose of the study in such a way that the potential participants will become truly curious about the results. In other words, they must understand that the project is about finding answers that will make their clinical work easier but that the project is not about proving a preconceived idea.

There are several different ways of recruiting data collecting participants. Clinicians interested in research can sign up for participation, for instance after information is given about the project at a general assembly or a professional meeting. A letter or an e-mail can be sent out to all the members of a professional association explaining the study’s aims and design, inviting those interested to sign up. Information must be short and to-the-point, including the purpose of the study, the potential benefits of the study, the tasks included for the participants, the number of patients needed and the time (number of minutes) that data collection will take per patient. This letter should be signed by the researcher and project officer, with contact addresses and telephone numbers. Recruitment can also be done by calling all the members of a target group personally and asking them if they are willing to participate, possibly after an explanatory letter has been sent out. If such calls are made, the logistics of the study can be explained during this phone conversation. If the clinicians are asked to make contact with the team to enquire more about the study, more detailed information can be provided at that point and they will not feel coerced to accept participation in the study during the conversation.

In clinics with a receptionist, the success of the data collection often depends on the involvement of the receptionist. Therefore, when the initial accept has been given by the chiropractor, the receptionist should usually be the contact person.

In order to obtain a representative sample of clinicians, it is important to give all individuals the opportunity to participate in the study. In reality, however, it will be necessary to make do with the more dedicated members of the profession

Regardless whether you attempt to recruit all registered chiropractors, all members of an association, participants at a political meeting, participants at an academic meeting or specially selected chiropractors only, it is unlikely that the data collecting chiropractors will be completely representative of the background population. It is more likely that there will be a bias towards the more academically inclined and/or those with a firm “belief”, those with an interest in research, and/or those who feel that they have the time to participate in the project. Only by collecting obligatory register data would it be possible to obtain a perfectly representative group of chiropractors.

There is usually no way of knowing whether a biased selection of clinicians will have an effect on the ultimate selection of patients, treatment and outcomes. Nevertheless, it is useful to obtain some demographic information on the data collecting chiropractors in relation to known factors that can be held up against the whole population of chiropractors. The national chiropractic association or registration board may have some demographic data that can be used for such purposes, such as age, sex, area of practice, school of graduation and years in practice.

#### Patients

The patients involved in a research project should be representative of the patients normally seen in a clinical setting. To avoid selection bias, it is important that the patients are enrolled consecutively in the study according to the inclusion criteria. Completely random selection of study participants requires a more complex procedure and is probably not realistic. During busy periods in the clinic and prior to major holidays, it is common that suitable patients are not invited into the study. This can be helped by selecting certain days or specific times of the day, when recruitment should take place, and allowing for some time to catch up, in case the inclusion procedure delays the usual clinic procedures somewhat.

Ideally, a record should be kept of all patients who are suitable for participation in the study and for all who were invited but declined participation. However, for practical reasons, this will probably not be feasible (as clinicians are busy). Therefore, it is primordial that clinicians are thoroughly informed of the importance of not selecting patients for inclusion in the study for some specific reason of their own but to let chance play the major role. The main reason for non-invitation into the study of an otherwise suitable study subject is acceptable if it is for administrative reasons and not for clinical reasons.

Patients should receive information about the study, both verbally and in writing, and should sign informed consent forms. This, of course, may make participation less attractive for patients who are in a hurry to get out to the parking metre or back to work. If this aspect is dealt with while the patient is waiting anyway (in the waiting room, at the reception area, in the treatment cubicle) patients in a hurry will not so easily be excluded from the study. Hopefully, this should keep non-participation to a minimum.

## Planning the study

### Meetings

The project group should meet with the researcher on several occasions to plan the study. These preparatory meetings could start with discussions of one or several clinical problems at hand. Clinicians are often naive about the lack of documentation for their clinical activities. At such a meeting, it might therefore be necessary to give an overview of a clinical problem and the lack of or conflicting evidence that exists, and the need for more or better knowledge could be pointed out. The study aims and objectives and the appropriate design and detailed method will then gradually take form. The researcher provides the methodological competence, whereas the project group has the knowledge needed for the practical considerations. Together, the group should be able to design a study that is relevant, methodologically sound and practically possible to perform. In addition, this process makes all the group members able to understand the study and defend the method chosen at all stages of the study process. When the preparation phase is over, the whole group should feel that it is ”their” project and that the best possible study is being carried out to answer the research question at hand. Ownership is really the key issue in order to keep this group working well.

It is important that these meetings are conducted in a pleasant environment and during joyful conditions. A positive social experience will make it easier for the project group to conduct the study.

### Designing and using questionnaires

During the process of planning, it will become apparent what information needs to be collected. If validated questionnaires exist, which is not always the case, it is preferable to use these. The researcher should provide advice on the available questionnaires, their validity, use and previous results. If need be, questionnaires can be designed by the project group.

Our experience with questionnaires is that if data are to be collected during the normal clinical encounter they should be short to enhance compliance. The easiest questionnaires have short and to-the-point questions with yes/no boxes to tick. It is easier to ask clinicians to collect information normally ascertained in the clinical setting than data not included in a standard consultation.

If possible, all information regarding one patient should be filled in on one piece of paper. Sometimes several questionnaires are needed (for example, one for the patient to fill in, two for the chiropractor; at baseline and another for follow up). In that case, these should be of different colours. It is easy to refer to the “blue” rather than the “inclusion” questionnaire when communicating with data collecting clinicians.

Patients’ anonymity must be retained at all times also for follow-up studies or whenever multiple questionnaires are required. If information is needed from one clinical occasion only, usually no patient identification is required, but if data are collected at several occasions, the clinician must be able to identify the patient on the questionnaire whilst data are collected, in order for the data to be correctly recorded for the “right” patient. In that case, we recommend that the patient’s last name and initial are written at the top or bottom of the questionnaire, at a dedicated space, to be cut off from the paper and destroyed when the data collection is complete.

When follow-up data are needed or when several questionnaires are used, it is necessary to code the questionnaires. We have used a set of three codes: one for the project group chiropractor, one for the data collecting chiropractor, and one for the patient. E.g. 010101 means project group chiropractor number 01, data collecting chiropractor number 01 and his patient number 01.

We keep the cost and effort for the data collecting clinicians to a minimum by providing them with a set of stamped and addressed return envelopes. If the patient has to fill in some information themselves, maybe of confidential type even to their treating chiropractor, also they should be provided with an individual envelope.

In order to have a good return rate on questionnaires filled out by chiropractors in relation to their patients, we have found that completing the questionnaires “in real time” (i.e. when the patient is in the clinic) is preferable. Questionnaires that patients are asked to fill in themselves should also be done whilst in the clinic. Otherwise, the patient may forget, mislay the questionnaire, or forget to return it to the clinic.

For logistic and ethical reasons, all questionnaires (at least in relation to the data collecting chiropractor) should be coded at, sent out from, and returned to the “research centre” to be handled by the project officer. This procedure minimizes errors, as all questionnaires are packed and coded the same way. The project group can assist in the packing of questionnaires and addressing envelopes. Only the project officer should have access to the “key” of codes, to ensure anonymity of the data collecting chiropractors and patients. When the project group gathers to analyse the data, no names of colleagues or patients should be available. And if, for some reason, the identity of a particular respondent becomes apparent, it must be explained to and imposed on the project group that this is highly confidential. The researchers must of course lead the way by being perfect role models in this respect.

### Ethical approval and considerations

All experimental research regarding human beings (and some other forms of data collection) need approval from a regional ethics committee. Normally, if no experimental treatment is carried out, the study will be regarded as a quality assurance project and the ethics committee will return the application with this comment or no ethics application may even be necessary. However, the rules differ from country to country and can also vary over time. It will therefore be necessary to make enquiries as to whether an approval is needed. If a computerized data file is created in which individuals can be identified, it is likely that a permit is needed also for this (data protection).

Whenever individuals can be identified, for example on questionnaires, or if questionnaires are anonymous but there is a list of names that can be related to the individual questionnaires, it is important that such information is kept safely locked up and that lists of names and corresponding codes not be kept together with the coded questionnaires.

It is a useful experience for the project group members to write an ethics application. It provides an opportunity to consider the ethical aspects of the study; the routines of using codes instead of names, the integrity of the patients and the safe storage of the data collected. In any case, time should be set aside to discuss ethical aspects with the project group, to impress upon them that this is a cardinal point in research.

### Pilot studies

If necessary, the project group can assist in the pilot testing of questionnaires and study routines in the clinical setting, i.e. in their own clinics. At a later stage, it will be essential for the members of this group to be able to answer questions from the data collecting clinicians regarding patients’ opinions, receptionists’ tasks and time spent on the study procedures. The data collecting clinicians will need information regarding time requirement before agreeing to participate, so this aspect is particularly important to settle before starting the main study.

When a new procedure or questionnaire is to be introduced, a pilot study in the clinical setting will provide the necessary measures of face validity and interpretability, and relevant changes can be made before the study commences. Pilot studies can also provide information on the feasibility of the targeted patient category, i.e. if it is common or not. This will decide the duration of the enrolment of patients in the study. Generally, we advice against collecting data on rare patient categories, as this takes too long and exhausts both project group members and clinicians.

Based on the results of the pilot study, the duration of data collection for the entire study can be estimated. However, it is a good idea to provide for more time than expected. Always estimate data collection duration by at least twice the calculated time. Various things will work against your study; clinicians go on holidays, their receptionist gets ill, their colleague quits and leaves, that particular patient type suddenly becomes rare. We also recommend that you never start a data collection period in the beginning of summer, when both clinicians and patients will soon go on holidays. Remember that also Christmas, Easter and other holidays have a tendency to disrupt routines.

### Workshops

The project group should help prepare for the recruitment of data collecting clinicians and for helping them through the data collection period. This is done through targeted telephone calls. These contacts require some skills beyond that of making an ordinary telephone call. Therefore the project group members should practice the phone calls (illustrated in the appendices) with another member of the group through role play. All possible “excuse”- scenarios should be tried and all possible encouraging comments should be invented and written down. This exercise will take a couple of hours and should be done with several rotations, i.e. each member of the group gets to train with several others, until the subject is exhausted.

## The data collecting chiropractors

### Telephone lists

Each project group member can be assigned 8–12 participating clinicians as “theirs”. The assigning is best done when the whole group is together at a planning session. If possible (easily done in a small association), friends can be assigned to each group member. This will make participation harder to refuse. People are much less willing to make up excuses when talking to a friend. Similarly, personal “enemies” should be avoided.

Each member of the project group will be responsible for making regular calls to “their” data collecting chiropractors. Thus, the same project group member will always call the same data collecting chiropractors unless later otherwise decided in conjunction with the researcher.

We suggest making telephone lists in Excel or on a similar paper version, with dates/weeks written in, so it is easy to keep track of who was called, when and what the outcome of the call was.

### Telephone calls and why they are important to ensure compliance

At every new step in the data collection, human inertia is likely to work against your project. The steps participating clinicians must go through are as follows:

A. Opening the envelope with the information material.

B. Having opened the envelope, actually reading it.

C. Having read it, also having understood it and considered the implications in the clinic.

D. Starting with the first case.

E. Continuing with the rest of the cases.

F. Returning the questionnaires.

The members of the project group will have to work diligently to overcome these obstacles on the way. This is done with systematic and frequent telephone calls. These phone calls follow a special “program” explained in detail in this manual and described in the additional files. Each of the obstacles of data collection should be addressed in an explanatory phone call.

Different types of clinicians will have to be treated differently. Basically, there are three types of data collecting clinicians, in relation to understanding the purpose and process of the study: Those who understand directly, those who understand after some extra explanations, and those who understand only after detailed information.

In relation to performance, there are four different types of participants: A small group who will do what they should do without any problems, a rather large group consisting of those who do what they should do after some prodding and/or with some delay; a somewhat smaller group which needs a lot of encouragement; and a small group consisting of those who fail to perform either in silence or despite many vivid assurances of active participation. Obviously, the phone calls need to be targeted to these different types; otherwise the minority group of non-performers can grow into a very large group indeed.

The calls should all be friendly, enthusiastic, patient, encouraging and professional. Each telephone conversation must be targeted to deal with the specific hurdle at hand. There is therefore no point in simply phoning the participants and asking how they are getting on, in a non-specific manner, as the answer invariably will be “good”. Then, as it becomes apparent to the participant that he is not performing, he will stop answering the phone calls and become inaccessible both by phone and e-mail.

## Telephone calls

### General points

The calls should be noted in the Excel sheet as: participating yes/no, next to the date called and the response to the call. If the person called is participating in the study, the date and time for the next scheduled telephone call should be noted. Similarly, all continued telephone encounters should be noted, until data have been successfully returned or the data collecting chiropractor has quit the study. Obviously, it is vital that all “appointments” for future calls are honoured. Any obstacles and the need for a new call in relation to each hurdle should be noted.

### Recruitment calls

Each potential participating clinician should be approached by one of the project group members in an introductory recruitment call, approximately 1 month before the study starts. The call should be outlined as described in Additional file 1.

### Complete telephone list

After this first round of telephone calls, there should be a list of who was contacted, of those refusing, those not reached and those agreeing to participate. Each project group member should receive a list of “their” data collecting chiropractors and the researcher should have the complete list of participants.

### First support call; responding to the hurdles: *have you received the material, opened the letter and read it?*

One week before the study starts, all participating clinicians are contacted again by their contact person. The call is described in Additional file 2.

If the clinician did not yet open the envelope, this call has to be repeated, preferably daily, until he/she does. If the clinician has opened the envelope, but has not looked at the material, this part of the conversation should be repeated in the same manner. Most chiropractors are not making this a priority in their daily practice, and therefore it is not uncommon that they have to be called several times. All calls should be noted for future reference and so should the date and time for the agreed next phone call.

### Following support calls; responding to the hurdles: *have you understood the study, have you started, are you proceeding?*

The following two or three (or as many as it takes!) weeks, each participant should be called to make sure they are proceeding as planned. If not, the call described in Additional file 2 is repeated. The important thing is to always call the clinicians back. It is fairly easy to ignore an e-mail or a letter, but a phone call (in particular from a friend) is difficult to ignore. The conversation will now concentrate on the data collection phase as described in Additional file 3.

It is, of course, everybody’s right to withdraw from an engagement such as this. No negative feelings should be placed on those who do; all you can say is that you are sorry. Further; some people are procrastinators, they postpone and provide all sorts of excuses. You need to be prepared and persevere with your calls. Sadly, you will notice that some promise a lot more than they deliver.

### Closing calls; responding to the hurdles: *have you finished and sent the questionnaires back to us?*

At the end of the data collection period, the participating clinicians should be reminded to collect data for the remaining patients and when this is done to send in the questionnaires. Some clinicians will not achieve the full number of patients and will have to return whatever data they have achieved at a specific deadline. This phone call is outlined in Additional file 4.

### Reporting and feedback

The project officer should obtain reports from all the project group members on a regular basis, at least every week. We recommend establishing a record also of this, and since this is quite time consuming, it is a good idea to exempt the project officer from being a contact person for data collecting clinicians.

Based on the feedback information from each group member, the project officer can provide feedback on the study progress to the entire group. Further, the project officer should keep track of patients recruited to the study by means of questionnaires/informed consent-forms coming in to the research centre. This will tell whether the data collecting clinicians are performing as planned. This feedback should then be forwarded to the responsible project group member, so he knows how successful his “team” is. In the case of non-performance by one (or several) data collecting chiropractors, the project officer is responsible for discussing this with the project group member, who is responsible for the failing data collector.

As soon as it becomes obvious that a data collecting clinician does not get started, despite the relevant encouraging calls, the project officer should be informed and the case discussed. Possibly, such a participant should be removed from the list. This requires a personal contact. Of course, the failing data collector should be told this in an unemotional manner. For example: “Judging by the number of patients you have enrolled in the study, it looks like you have a busy schedule or that you may not see the right patient type for this project at the moment. This may not be the best time for you to participate in a study like this. What do you say, is it better to remove you from our list?”

## Analysis and report preparation

### Initial data inspection

The project group should meet with the researcher for a first inspection of the data. All the raw data should be available to the group, literally to the touch. Together, the group can count the number of questionnaires received, and complete the first data cleaning; i.e. decide which questionnaires are too incomplete to be included. These decisions should be written down for future reference and for the final report/research article.

### Summative and descriptive analysis

If feasible (up to around 1000 included patients), data can be entered by hand onto large spread sheets. Each pair of project group members gets a batch of questionnaires, and is made responsible for summing up one variable at a time, each in the pair checking the quality of the data entry and counts. The estimate count for each variable obtained by this pair is then reported to an appointed “writer” for example on a board for all to see; the number of positives, negatives and missing. In this way, all the data are added up into one final estimate. This is suitable for all descriptive data and can be done also for some simple cross-tabulations. The method provides a feel for the data not otherwise provided in a computer entered analysis. Further, the members of the project group get to see all the errors possible when filling out a questionnaire, useful knowledge for future projects. These errors should also be noted for reference when designing questionnaires in future studies.

### Computerized statistical analysis

Obviously, when the object of the study is to investigate associations or interactions between variables, statistical computer software needs to be used. In such a case, data can be entered by one or several members of the groups with the usual quality assurance methods (double data entry or random checks). The statistical methods can be explained to the group by the researcher or a statistician, in such a way that they understand why they are used and, in particular, how the results are interpreted.

### Data interpretation

The results of the analysis should then be presented to the project group, and form the basis for discussions within the group. Are the results as expected? Why or why not? What are the clinical implications? Are any further analyses suitable? These discussions are the base for the final report or research article. It is important to include the project group at this stage, as this is when the fun starts. This is their moment of reward, after all the hard work and tedious phone calls.

### Writing the research report

The project group should, after the interpretation of the results, design the crude outline of the scientific study report. Aided by the researcher, the group can decide the outline of the background, methodology, results and discussion sections. Examples from good and bad research reports can be used to make this easier. The project group can also divide the report between them, some members writing the method section, some the results, and some the discussion. The researcher or project officer will ultimately have the responsibility of putting the fragments together and editing the complete manuscript, after which the project group should proofread and comment. Their active participation during this stage will make the final report less technical and more easily understood by ordinary clinicians. This is important, because unless the research article is easily accessible to clinicians, the information it contains will not enter their conceptual world and useful information will not become generally implemented.

## Management

### Project group

In the beginning, it might be a good idea to include more participants in the project group than needed, as it is not uncommon that in a group of 5–8, one or two will drop out when they realize that research requires consistency and tedious tasks. Persons with the best personalities to do this type of work are those who are conscientious and socially gifted. It is always a good idea to include in the group at least one realistically critical person, who finds the weak points in the study design and in the data collection process. It is also helpful to have access to somebody who is interested in computer layout, for the successful design of questionnaires.

Individuals less well suited for this work are those who find it difficult to work together in a team and to conform. Luckily, they will soon single themselves out by their constant need to express contrary opinions. Such individuals are more comfortable working on their own and should not be convinced to stay on in the project when they start gliding out.

Sometimes one or several members of the project group do not perform according to the protocol during the crucial data collecting phase. This can take one of two forms: Phone calls are not made or phone calls are not made according to the agreed procedure. This will become apparent early in the process from the lower response rate from “their” data collection practitioners. When the project officer notices such an anomaly, it will be necessary to ask tactfully if one (or several) data collecting clinician presents a problem. In that case, it might be best to move this/these participants on to another member of the project group, who might be more successful in establishing a positive contact. It will sometimes be necessary for the project officer to take over the task of managing some or all of these data collecting chiropractors, as otherwise the whole study is jeopardized.

If however, the problem seems to lay with the project group member, the project has a problem. Either the failing group member must be made to understand that the agreed upon procedure must be followed or asked to leave the group. If this is difficult, then at least, ensure that this person is not enrolled for your next study. In the meantime the project officer must take over all or some of the failing group member’s tasks.

### Data collecting participants

During recruitment, we suggest that people who are consistently difficult to reach are left out of the study, as they tend to be unavailable throughout the whole study period and therefore, usually will be non-compliant.

Obviously, there are those who are less suitable participants than others, which needs to be ascertained during the recruitment phone call outlined in Additional file 1. Newly graduated chiropractors who are starting up their own clinic may not have enough patients to be able to provide the required number of study subjects. Extremely busy practitioners will not be able to carry through with their commitment, because their practice procedures usually do not allow for any flexibility.

The odd person will want to change the study protocol, and if the comments are relevant should be listened to and perhaps invited to participate in the project group. However, if the comments are uninformed and the person obviously does not trust the researcher’s competence, he or she should be excluded from the data collection group, if sabotage of data seems likely

## Feedback

### Project group

The project group meets to discuss the results of the study and to plan the final manuscript/ publication. The final discussions should also include the issue of clinical applicability and the next possible steps forward. We have found that many times the wrap up of one study is the start of the next. The project group members’ formal reward for participating is that they get their name on a scientific publication. However, the experience of doing practice based research and finding the answers to clinically relevant questions is rewarding in itself and can be a source of great joy for the group members.

### Data collecting participants

Making the results of the study known to the clinicians involved in the data collection is important if their participation is wanted in future studies. In this matter, one cannot rely on clinicians reading the resulting scientific publication. We suggest that the report/ publication be sent directly to all who collected data, as well as to the members of their national association. One can include an explanatory letter (stating the results of the study in a couple of sentences) for those who are not interested in reading the full paper. Such a letter has double intentions. First the result should reach those who can implement it, i.e. the clinicians. Second, credit is given to those participating, both members of the project group and the data collecting chiropractors.

We also recommend that the researcher or project officer presents the results at a general assembly to alert all those affected by the findings. This is also an excellent opportunity to praise those donating their time and effort, and to try to awake an interest in others to participate in the next study.

To acknowledge the data collecting clinicians, after each study is completed, we provide them with a diploma. This diploma reads: “The chiropractor in this clinic is contributing to making chiropractic treatment evidence based through active participation in research”. The reference of the ensuing publication could also be noted in the diploma text. The idea is that this diploma should hang in the involved clinics making patients aware that their chiropractor is involved in research. For some of our participating clinicians, the fact that they can put “participation in research” on their CV has been an advantage when applying for reimbursement plans through insurance and other funding schemes.

### Scientific community

Of course, the results of any study should be made known to the scientific community as a publication in a peer reviewed journal and as presentations at scientific conferences. In fact, it would be unethical not to do so.

## Discussion

To our knowledge, this is the first manual describing the procedures for conducting a multicentre clinical study in the chiropractic setting. As the method has been tried several times, we believe it to be worthwhile and it may be of value to other researchers in the clinical field.

### The clinical perspective

By going through the processes outlined in this manual, clinical studies in multicentre settings are possible and it should be feasible to obtain answers to a number of research questions. In other words, we will have obtained knowledge of some clinically relevant issues that will benefit our patients.

The described procedure will enlighten the project group members as to the importance of rigours of protocol and the hard work of ensuring compliance among clinicians. Moreover, at the end of execution of a study like this, they will be knowledgeable in the methodology chosen, a valuable experience for any clinician expected to read and evaluate research articles as part of their everyday practice.

### The research perspective

Because of the involvement of several “ordinary” field practitioners and patients, and because the study is taking place in the usual clinical setting, it is likely that the research conducted in this manner is not only relevant but that the study participants are representative of the “typical” clinical population and that the results are fairly generalizable. In a well conducted project one can expect the data to be trustworthy. When the various tasks are divided between many people, who are willing to donate their time to a good cause, the project will also have been cheap to conduct. The end result will, hopefully, be a clinically relevant and interesting publication, a stepping stone towards a better understanding of the clinical work carried out every day in ordinary practice.

### Limitations

The procedures outlined in this manual are not scientifically tested against other procedures, they are merely empirically anchored.

Further, they are tested mainly amongst chiropractors in the Nordic countries. In these countries, the chiropractic associations are small and colleagues often know each other. Therefore, participation may be easier to achieve. Experiences from the international study [13] were that the project group members approached their task differently, some obviously not willing to follow instructions, possibly for cultural reasons, whereas others did. The response rates in the different countries reflected these differences. Hence, it is likely that cultural differences play a role in relation to this type of study set-up.

### Future projects

When the study is completed, the project members will know which clinicians to contact for future studies (a convenience sample). Make a list of these! This will be a group of people who are interested in research, willing to donate the necessary time and effort, and who will be competent co-workers when you invite them in the future. Rather, they are likely to be more compliant in future studies with a need for fewer prompting calls.

## Conclusions

We have described a method of conducting clinical studies in the chiropractic setting including several steps to ensure good compliance. We believe that our experience of how to set up this type of studies i.e. the organisation of collaborators, the involvement of clinicians and the procedures outlined in this manual should be made available to others as it seems to be a feasible way of obtaining high compliance and to perform relevant clinical research. This method may be expanded to other clinical fields as well.

## Competing interests

The authors declare that they have no competing interests.

## Authors’ contributions

Both authors have developed the method described herein, and have written and approved the final manuscript.

## Supplementary Material

Additional file 1 The recruitment call. Click here for file

Additional file 2 The first support call. Click here for file

Additional file 3 Following support calls. Click here for file

Additional file 4 The closing calls. Click here for file

## References

[B1] Leboeuf-Yde C Axen I Ahlefeldt G Lidefelt P Rosenbaum A Thurnherr T The types and frequencies of improved nonmusculoskeletal symptoms reported after chiropractic spinal manipulative therapy J Manipulative Physiol Ther 1999 22 9 559 564 10.1016/S0161-4754(99)70014-X 10626697

[B2] Leboeuf-Yde C Hennius B Rudberg E Leufvenmark P Thunman M Chiropractic in Sweden: a short description of patients and treatment J Manipulative Physiol Ther 1997 20 8 507 510 9345678

[B3] Leboeuf-Yde C Gronstvedt A Borge JA Lothe J Magnesen E Nilsson O Rosok G Stig LC Larsen K The nordic back pain subpopulation program: demographic and clinical predictors for outcome in patients receiving chiropractic treatment for persistent low back pain J Manipulative Physiol Ther 2004 27 8 493 502 10.1016/j.jmpt.2004.08.001 15510092

[B4] Axen I Rosenbaum A Robech R Wren T Leboeuf-Yde C Can patient reactions to the first chiropractic treatment predict early favorable treatment outcome in persistent low back pain? J Manipulative Physiol Ther 2002 25 7 450 454 10.1067/mmt.2002.126473 12214186

[B5] Axen I Rosenbaum A Robech R Larsen K Leboeuf-Yde C The Nordic back pain subpopulation program: can patient reactions to the first chiropractic treatment predict early favorable treatment outcome in nonpersistent low back pain? J Manipulative Physiol Ther 2005 28 3 153 158 10.1016/j.jmpt.2005.02.007 15855901

[B6] Axen I Jones JJ Rosenbaum A Lovgren PW Halasz L Larsen K Leboeuf-Yde C The Nordic Back Pain Subpopulation Program: validation and improvement of a predictive model for treatment outcome in patients with low back pain receiving chiropractic treatment J Manipulative Physiol Ther 2005 28 6 381 385 10.1016/j.jmpt.2005.06.008 16096036

[B7] Leboeuf-Yde C Axen I Jones JJ Rosenbaum A Lovgren PW Halasz L Larsen K The Nordic back pain subpopulation program: the long-term outcome pattern in patients with low back pain treated by chiropractors in Sweden J Manipulative Physiol Ther 2005 28 7 472 478 10.1016/j.jmpt.2005.07.003 16182020

[B8] Leboeuf-Yde C Gronstvedt A Borge JA Lothe J Magnesen E Nilsson O Rosok G Stig LC Larsen K The Nordic back pain subpopulation program: a 1-year prospective multicenter study of outcomes of persistent low-back pain in chiropractic patients J Manipulative Physiol Ther 2005 28 2 90 96 10.1016/j.jmpt.2005.01.010 15800507

[B9] Axen I Jensen IB Eklund A Halasz L Jorgensen K Lange F Lovgren PW Rosenbaum A Leboeuf-Yde C The Nordic Maintenance Care Program: when do chiropractors recommend secondary and tertiary preventive care for low back pain? Chiropr Osteopat 2009 17 Jan 1 19161611 10.1186/1746-1340-17-1 PMC2633010

[B10] Malmqvist S Leboeuf-Yde C Ahola T Andersson O Ekstrom K Pekkarinen H Turpeinen M Wedderkopp N The Nordic back pain subpopulation program: predicting outcome among chiropractic patients in Finland Chiropr Osteopat 2008 16 13 10.1186/1746-1340-16-13 18992154 PMC2588613

[B11] Leboeuf-Yde C Hennius B Rudberg E Leufvenmark P Thunman M Side effects of chiropractic treatment: a prospective study J Manipulative Physiol Ther 1997 20 8 511 515 9345679

[B12] Leboeuf-Yde C Rosenbaum A Axen I Lovgren PW Jorgensen K Halasz L Eklund A Wedderkopp N The Nordic Subpopulation Research Programme: prediction of treatment outcome in patients with low back pain treated by chiropractors - does the psychological profile matter? Chiropr Osteopat 2009 17 1 14 10.1186/1746-1340-17-14 20042095 PMC2807423

[B13] Leboeuf-Yde C Pedersen EN Bryner P Cosman D Hayek R Meeker WC Shaik J Terrazas O Tucker J Walsh M Self-reported nonmusculoskeletal responses to chiropractic intervention: a multination survey J Manipulative Physiol Ther 2005 28 5 294 302 discussion 365–296 10.1016/j.jmpt.2005.04.010 15965403

[B14] van den Hoogen HJ Koes BW van Eijk JT Bouter LM Deville W On the course of low back pain in general practice: a one year follow up study Ann Rheum Dis 1998 57 1 13 19 10.1136/ard.57.1.13 9536816 PMC1752458

[B15] Gheldof EL Vinck J Vlaeyen JW Hidding A Crombez G Development of and recovery from short- and long-term low back pain in occupational settings: a prospective cohort study Eur J Pain 2007 11 8 841 854 10.1016/j.ejpain.2006.12.012 17314055

[B16] Carey TS Garrett JM Jackman AM Beyond the good prognosis. Examination of an inception cohort of patients with chronic low back pain Spine 2000 25 1 115 120 10.1097/00007632-200001010-00019 10647169

