# Supplementary material for: Conducting practice-based projects among chiropractors: a manual
Source: Chiropr Man Therap. 2013 Feb 1;21:8. doi: 10.1186/2045-709X-21-8 (PMC3577479; doi:10.1186/2045-709X-21-8)
Supplement: Additional file 1 — The recruitment call. [file 2045-709X-21-8-S1.docx]

ADDITIONAL FILE 1

The recruitment call.

- Hello, this is ......I am calling about a research project at .....Have you got a few minutes?
- No? I understand. When will be a good time to call you back?

OR

- Yes? Great! I would like to briefly tell you about this project: I am working in a group lead by ...
- The aim of the study is....
- and we are going to collect data in the clinics throughout ....and we are starting in the month of .....
- What we would like your help with, is collecting data on 10 patients.....with.....
- It will take up ....minutes of your time per patient and
- you will have to fill in questionnaires regarding .... on the 1^st^ and .....visit.
- Do you think that this is something you would like to participate in? Is it feasible, with your workload and clinical setting, that you will be able to collect data as outlined?
- No? That is completely OK, thanks for your time!

OR

- Yes? Thank you, we will send you the necessary information and material, and I will get in touch with you during....in due time before the study starts.
- What time is usually the best to call you (day and time of day)?
- Here is my telephone number if you have any questions. I will be your contact person.
- OK then, thank you once again. I will be calling you on......at...... Bye!
