# Supplementary material for: Conducting practice-based projects among chiropractors: a manual
Source: Chiropr Man Therap. 2013 Feb 1;21:8. doi: 10.1186/2045-709X-21-8 (PMC3577479; doi:10.1186/2045-709X-21-8)
Supplement: Additional file 2 — The first support call. [file 2045-709X-21-8-S2.docx]

ADDITIONAL FILE 2

The first support call.

- Have you received the material yet?
- No? I’ll send you a new set!

OR

- Yes? Good! Did you look at the material yet?
- No? I’ll call tomorrow then, when you’ve had a chance to look through it. What will be a good time?

OR

- Yes? Let’s go through the study procedures and questionnaires:
  - The patients should have the symptom /diagnosis of...
  - They should be in the ages of...
  - They should be new patients or....
  - They should be able to understand the language of....
  - When the patient comes in, you fill in the yellow form. As you can see, the information we want is.....
  - On the first visit, the patient is also asked to fill in the green form, and put it in the enclosed envelope. We would like your receptionist to mail these to us on a daily basis.
  - Then, when the patient returns on the ...visit, you fill in the blue form..
- I’ll call you next week to hear how you’re getting along. Please don’t hesitate to call me if you have any questions. Good luck!
