# Supplementary material for: Conducting practice-based projects among chiropractors: a manual
Source: Chiropr Man Therap. 2013 Feb 1;21:8. doi: 10.1186/2045-709X-21-8 (PMC3577479; doi:10.1186/2045-709X-21-8)
Supplement: Additional file 3 — Following support calls. [file 2045-709X-21-8-S3.docx]

ADDITIONAL FILE 3

Following support calls.

- Did you start to collect data yet?
- No? Do you have any questions? It is important to get started this week! Do you think you will get started tomorrow? Good, I’ll call you tomorrow evening (or the decided day)...

OR

- Yes? Excellent! How is it working out? Are targeted patients coming in? Do you have any questions regarding any of the questionnaires/ procedures?
- Thank you for participating! Keep up the good work!
