# Supplementary material for: Conducting practice-based projects among chiropractors: a manual
Source: Chiropr Man Therap. 2013 Feb 1;21:8. doi: 10.1186/2045-709X-21-8 (PMC3577479; doi:10.1186/2045-709X-21-8)
Supplement: Additional file 4 — The closing calls. [file 2045-709X-21-8-S4.docx]

ADDITIONAL FILE 4

The closing calls.

- Did you finish collecting data?
- No? I’ll call you back next week to hear how you’re getting on. It is important that you collect the final cases now, we need to finish up.

OR

- Yes? Great! Now all you have to do is to put the questionnaires in the provided envelope and mail it to us. Thank you so much!

OR

- No? Well, we have met the time limit for data collection, so I would like you to send in the data that you have collected. You just put the questionnaires in the provided envelope and mail it to us. Thank you so much!
